# Optical Coherence Tomography Evaluation of Carotid Artery Stenosis and Stenting in Patients With Previous Cervical Radiotherapy

**DOI:** 10.3389/fnins.2022.861511

**Published:** 2022-04-27

**Authors:** Xiaohui Xu, Feihong Huang, Xuan Shi, Rui Liu, Yunfei Han, Min Li, Fang Wang, Qingwen Yang, Wusheng Zhu, Ruidong Ye, Xinfeng Liu

**Affiliations:** ^1^Department of Neurology, Affiliated Jinling Hospital, Medical School of Nanjing University, Nanjing, China; ^2^Department of Neurology, The First School of Clinical Medicine, Jinling Hospital, Southern Medical University, Nanjing, China; ^3^Department of Neurology, Guilin People’s Hospital, Guilin, China; ^4^Department of Neurology, Jiangsu Province Hospital of Chinese Medicine, Nanjing, China; ^5^Department of Neurology, Jinling Hospital, Southeast University School of Medicine, Nanjing, China; ^6^Division of Life Sciences and Medicine, Stroke Center & Department of Neurology, The First Affiliated Hospital of University of Science and Technology of China (USTC), University of Science and Technology of China, Hefei, China

**Keywords:** carotid artery stenosis, radiotherapy, stenting, optical coherence tomography, morphological characteristics

## Abstract

**Objectives:**

Cervical radiotherapy can lead to accelerated carotid artery stenosis, increased incidence of stroke, and a higher rate of in-stent restenosis in irradiated patients. Our objective was to reveal the morphological characteristics of radiation-induced carotid stenosis (RICS) and the stent–vessel interactions in patients with previous cervical radiotherapy by optical coherence tomography (OCT).

**Materials and Methods:**

Between November 2017 and March 2019, five patients with a history of cervical radiotherapy were diagnosed with severe carotid artery stenosis and underwent carotid artery stenting (CAS). OCT was conducted before and immediately after the carotid stent implantation. Two patients received OCT evaluation of carotid stenting at 6- or 13-month follow-up.

**Results:**

The tumor types indicating cervical radiotherapy were nasopharyngeal carcinoma (*n* = 3), cervical esophageal carcinoma (*n* = 1), and cervical lymphoma (*n* = 1). The median interval from the radiotherapy to the diagnosis of RICS was 8 years (range 4–36 years). Lesion characteristics of RICS were detected with heterogeneous signal-rich tissue, dissection, and advanced atherosclerosis upon OCT evaluation. Post-interventional OCT revealed 18.2–57.1% tissue protrusion and 3.3–13.8% stent strut malapposition. Follow-up OCT detected homogeneous signal-rich neointima and signal-poor regions around stent struts. In the patient with high rates of tissue protrusion and stent strut malapposition, the 6-month neointima burden reached 48.9% and microvessels were detected.

**Conclusion:**

The morphological features of RICS were heterogeneous, including heterogeneous signal-rich tissue, dissection, and advanced atherosclerosis. Stenting was successful in all 5 patients with severe RICS. One patient, with high rates of tissue protrusion and stent strut malapposition immediately after stenting, received in-stent neointimal hyperplasia at a 6-month follow-up.

## Introduction

Radiotherapy is an effective treatment for head and neck cancer and dramatically extends life expectancy. However, there is a major issue that radiation-induced cerebral vasculopathy may occur during the long-term follow-up, such as cerebrovascular stenosis or occlusion (Twitchell et al., 2018). It is reported that the cumulative incidence of moderate (>50%) carotid stenosis in the first, second, and third years following head and neck radiotherapy is 4, 12, and 21%, respectively (Texakalidis et al., 2020). The mechanism of radiation-induced carotid stenosis (RICS) has been undetermined in the literature. Relevant hypotheses include radiation insult to the intima-media (accelerated atherosclerosis) and injury to the vasa vasorum in the adventitia (a distinct disease entity) (Plummer et al., 2011). To date, the histopathological findings of RICS based on small cases are various, including ulcerating atherosclerosis with calcification, periarterial fibrosis, damage to the vasa vasorum, necrotizing vasculitis, thrombosis, and transmural fibrosis (Levinson et al., 1973; Atkinson et al., 1989; Zidar et al., 1997; Tonomura et al., 2018).

Furthermore, the incidence of stroke significantly increases after cervical radiation and the relative risk of stroke reaches 5.6 in irradiated patients (Gujral et al., 2014). To reduce the risk of stroke, carotid artery stenting (CAS) is performed in patients with severe RICS. Nevertheless, the long-term outcomes after CAS show a markedly higher rate of in-stent restenosis in patients with previous radiotherapy, reaching 25.7% in 2 years (Yu et al., 2014). To the best of our knowledge, the stent–vessel relationship immediately after stenting and the pattern of in-stent neointimal hyperplasia in patients with RICS have not been studied yet. Overall, the morphological features of RICS and the stent–vessel interactions in patients with previous cervical radiotherapy remain to be examined. With the help of the intravascular optical coherence tomography (OCT) technique, we may achieve that aim.

Optical coherence tomography is a vessel wall imaging technique with the highest resolution (10–20 μm) and has been known as an optical biopsy technique (Tearney et al., 2012). It has been applied to evaluate the carotid atherosclerotic plaque and the stent–vessel interactions since 2010 (Yoshimura et al., 2010, 2011; Jones et al., 2012, 2014; Setacci et al., 2012; Given et al., 2013; Liu et al., 2015, 2019; Funatsu et al., 2020; Shi et al., 2020). The purpose of this preliminary study was to apply OCT to reveal the morphological characteristics of RICS and the stent–vessel interactions in patients with previous cervical radiotherapy.

## Materials and Methods

### Study Design and Patient Selection

Between November 2017 and March 2019, five patients (≥18 years old) with severe internal carotid artery (ICA) stenosis (70–90% diameter reduction) detected by digital subtraction angiography (DSA) and previous cervical radiotherapy were enrolled for pre-interventional OCT evaluation. Immediately after stenting, 4 patients underwent OCT evaluation. Since the patient experienced vasospasm and the blockage of the cerebral protection device, 1 patient did not receive post-interventional OCT evaluation. Only two patients underwent 6- or 13-month follow-up OCT evaluation since the other three patients refused another OCT evaluation due to the relatively expensive fee and the fear of invasive examination. Patients were from Jinling hospital in Nanjing, China. The study protocol was approved by the hospital’s ethics committee. Written informed consents were obtained from all patients. The relevant clinical and radiologic data were reviewed.

### Optical Coherence Tomography Image Acquisition and Analysis

The intravascular frequency-domain OCT imaging system (ILUMIEN OPTIS, St. Jude Medical, Massachusetts, United States) and 2.7F OCT imaging catheters (Dragonfly Duo, Abbott Medical, Massachusetts, United States) were used for OCT image acquisition. With an 8F guide catheter placed in the common carotid artery (CCA), a cerebral protection device [FilterWire (Boston Scientific, Massachusetts, United States), Emboshield NAV6 (Abbott Vascular, California, United States), or ANGIOGUARD (Cordis Corporation, Florida, United States)] was advanced through the guide catheter and positioned in the ICA C1 segment. An OCT imaging catheter was then carefully advanced over the guidewire of the cerebral protection device and navigated through the carotid artery lesion. During the blood clearance by the automatic injection of 20 ml of undiluted iodixanol 320 (GE Healthcare Ireland Limited, County Cork, Ireland) with a velocity of 10 ml/s through the 8F guide catheter, the light mirror of the OCT imaging catheter was helically pulled back (18 or 36 mm/s) to get a series of cross-sectional OCT images of the vessel wall (Jones et al., 2014; Liu et al., 2015; Shi et al., 2020). OCT image acquisition was repeated immediately after stenting and during the follow-up DSA examination. The OCT imaging catheter was introduced into the ICA over a 0.014-inch microwire during the follow-up DSA examination.

Optical coherence tomography images were analyzed independently by two investigators (XX and FH) with extensive experience in reviewing OCT images. OCT images within 10 mm distal and proximal to the minimum lumen were assessed. The image was considered non-analyzable if the assessment of a continuous 270° arc was impaired by the intraluminal blood (Jones et al., 2014). Before quantitative measurement, manual OCT calibration was performed along with the entire pullback. Cross-sectional OCT images were analyzed at 0.1- or 0.2-mm intervals. The rates of tissue protrusion and stent strut malapposition were analyzed at 1-mm intervals (de Donato et al., 2013; Liu et al., 2015). The rates of tissue protrusion were calculated in the slice-based analysis, and the rates of stent strut malapposition were in the strut-based analysis.

Qualitative and quantitative OCT evaluations were performed precisely based on previously published criteria (Prati et al., 2010; Tearney et al., 2012). Lipid-rich plaque was defined as plaque with lipid content occupying more than one quadrant (Tearney et al., 2012). The calcific nodule was defined as single or multiple regions of calcium that protruded into the lumen (Tearney et al., 2012). A thin fibrous cap was present if the fibrous cap thickness was less than 65 μm (Tearney et al., 2012). Plaque rupture was defined as the discontinuity of the fibrous cap and the cavity formation, with or without a superimposed thrombus (Prati et al., 2010; Tearney et al., 2012). A thrombus was defined as a mass (≥250 μm) attached to the luminal surface or floating within the lumen (Jang et al., 2005; Tearney et al., 2012). Microvessels or neovascularization was defined as signal-voiding tubular structures (50–300 μm) present on at least three consecutive cross-sectional frames (Takano et al., 2009; Prati et al., 2010; Li et al., 2020). Dissection was defined as the presence of an intimomedial flap producing a double-lumen or an intramural hematoma formation (Alfonso et al., 2012). Intramural hematoma can be defined as a relatively homogeneous signal-rich material with variable attenuation separating the intima from the outer vessel wall (Alfonso et al., 2012). Tissue protrusion was defined as the prolapse of tissue into the lumen between adjacent stent struts (Tearney et al., 2012). It was divided into 3 groups, namely, smooth protrusion, irregular protrusion, and protrusion with attenuation (Funatsu et al., 2020). The distance from the surface of the stent strut to the lumen contour was measured. A stent strut was classified as “malapposed” (distance > 200 μm), “well apposed” (distance 10–200 μm), or “embedded” (distance < 10 μm) (de Donato et al., 2013; Liu et al., 2015). Follow-up OCT image with the minimum lumen was selected to measure the neointima burden. It was calculated as (stent area – lumen area)/stent area × 100% (Tearney et al., 2012).

### Carotid Artery Stenting Procedure and Quantitative Angiography Analysis

The CAS procedure was performed through the femoral approach through guide catheters. All patients underwent systemic anticoagulation with 4,000 IU unfractionated heparin after femoral artery puncture and an additional 2,000 IU/h. Pre-dilatation of the carotid lesion with a 4.0–5.0-mm balloon catheter was performed after OCT image acquisition. Stenting was then performed with one of the following open-cell stents: Acculink (Abbott Vascular, California, United States), Precise (Cordis, Florida, United States), and Protégé (Medtronic, Minnesota, United States). The selection of the stent diameter and length was decided by our experienced interventional neurologists according to the lesion feature. Post-dilatation was performed with a 5.0–6.0-mm balloon catheter when the remaining stenosis was > 30%.

The degree of diameter stenosis, residual stenosis, and in-stent stenosis was calculated accurately according to the North American Symptomatic Carotid Endarterectomy Trial criteria (Barnett et al., 1998). Technical success was defined as the residual stenosis < 30% on DSA. In-stent restenosis was defined as ≥ 50% stenosis within or at the edge of the stent. Angiograms and OCT images used co-registration on the basis of the landmarks, such as the bifurcation of the CCA and the stent edge, to ensure that they were at identical sites.

## Results

### Patient Characteristics

A total of 5 male patients [mean (standard deviation) age, 64.2 (8.5) years] with previous cervical radiotherapy underwent OCT evaluation of carotid artery stenosis successfully (Table 1). The tumor types indicating cervical radiotherapy were nasopharyngeal carcinoma (NPC) (*n* = 3, 60%), cervical esophageal carcinoma (*n* = 1, 20%), and cervical lymphoma (*n* = 1, 20%). The radiation dose was 70 Gy in three patients. The median interval from the radiotherapy to the diagnosis of RICS was 8 years (range 4–36 years). Interestingly, all patients had not more than one risk factor for atherosclerosis but 60% of them had severe bilateral carotid disease (70–100% diameter reduction). Except for one patient diagnosed with severe right coronary artery stenosis, all patients were free of coronary artery disease and peripheral artery disease.

**TABLE 1:** Demographic, clinical, and radiological features of five patients with a history of cervical radiotherapy.

Case No./Age (years)/Sex	Tumor type	Treatment	Radiation to diagnosis interval (years)	Risk factors for atherosclerosis	Carotid artery stenosis	CAD	PAD
1/61/male	Nasopharyngeal carcinoma	Radiation (70 Gy) and chemo	4	None	LICA 70%	RCA 95%	None
2/53/male	Nasopharyngeal carcinoma	Radiation (70 Gy) and chemo	8	Hypertension	LICA 80% LECA 100%	None	None
3/75/male	Nasopharyngeal carcinoma	Radiation	36	Smoking	LICA 90% LECA 100% RCCA 100%	None	None
4/70/male	Cervical esophageal carcinoma	Radiation (70 Gy) and chemo	4	None	RICA 70% LICA 100%	None	None
5/62/male	Cervical lymphoma	Radiation and chemo	15	Hypertension	LCCA 70% RCCA 100%	None	None

*Risk factors for atherosclerosis include smoking, obesity, diabetes mellitus, hypertension, and hypercholesterolemia.*

*CAD, coronary artery disease; LCCA, left common carotid artery; LECA, left external carotid artery; LICA, left internal carotid artery; PAD, peripheral artery disease; RCA, right coronary artery; RCCA, right common carotid artery; RICA, right internal carotid artery.*

### Digital Subtraction Angiography and Optical Coherence Tomography Analysis

As for the evaluated carotid artery lesion, the mean degree of diameter stenosis was 76% (range 70–90%) on DSA and the mean minimum lumen area was 3.10 mm^2^ (range 1.57–5.23 mm^2^) on OCT (Table 2). The morphological characteristics of RICS in three NPC patients were different (Cases 1–3). In the NPC patient with a 4-year interval from radiotherapy, heterogeneous signal-rich tissue was observed in consecutive 30 frames (6 mm) and occupied almost three-quarters of the vessel at the minimum lumen (Case 1). Atypical lesion such as dissection in the ICA C1 segment was detected in the other two NPC patients with longer time intervals (Cases 2 and 3). In the NPC patient with an 8-year interval, multiple cavity formations were present besides the dissection (Case 2). In the NPC patient at a 36-year interval, advanced atherosclerosis such as ruptured lipid-rich plaque, ruptured calcific nodule, and thrombosis coexisted with the dissection (Case 3). The morphological characteristics of RICS in patients with esophageal carcinoma or lymphoma were similar to atherosclerosis (Cases 4 and 5). Lipid-rich plaque was present in both cases. Neovascularization and thrombosis were revealed in the patient with longer time intervals (Case 5).

**TABLE 2:** DSA and OCT features of radiation-induced carotid stenosis and the stent-vessel interactions.

Case no./age (years)/Sex	Evaluated vessel site	DS, %, DSA	MLA, mm^2^, OCT	Lesion features, OCT	Treatment	RS, %, DSA	Stent-vessel relationship, OCT	Tissue protrusion, %, OCT	Stent strut malapposition, %, OCT	Follow-up in-stent restenosis, DSA	Follow-up OCT	Neointima burden, %, OCT
1/61/male	LICA sinus	70%	3.46	Heterogeneous signal-rich tissue.	Angioplasty and Stenting	20%	Smooth tissue protrusion.	35.7%	5.4%	None	NA	NA
2/53/male	LICA C1	80%	2.20	Dissection; multiple cavity formations.	Angioplasty and Stenting	20%	Smooth tissue protrusion; small dissection.	28.6%	3.3%	NA	NA	NA
3/75/male	LICA C1	90%	1.57	Dissection; ruptured lipid-rich plaque; ruptured calcific nodule; thrombosis.	Angioplasty and Stenting	20%	NA	NA	NA	None	13-month homogeneous neointima; signal-poor regions around struts.	31.6%
4/70/male	RICA sinus and C1	70%	5.23	Lipid-rich plaque.	Angioplasty and Stenting	20%	Smooth and irregular tissue protrusion; small dissection.	57.1%	13.8%	None	6-month homogeneous neointima; neovascularization; signal-poor regions around struts.	48.9%
5/62/male	Bifurcation of LCCA	70%	3.03	Lipid-rich plaque; neovascularization; thrombosis.	Angioplasty and Stenting	20%	Smooth tissue protrusion.	18.2%	5.6%	NA	NA	NA

*DS, diameter stenosis; DSA, digital subtraction angiography; LCCA, left common carotid artery; LICA, left internal carotid artery; MLA, minimum lumen area; NA, not available; OCT, optical coherence tomography; RICA, right internal carotid artery; RS, residual stenosis.*

All patients received balloon pre-dilatation and carotid stenting. The rate of technical success was 100% on DSA. Four patients underwent OCT examination immediately after stenting to evaluate the stent–vessel relationship in RICS patients (Cases 1, 2, 4, and 5). A total of 67 cross-sectional OCT images [mean (standard deviation), 17 (5)] were analyzed to assess the rates of tissue protrusion and stent strut malapposition. The mean rates of tissue protrusion and stent strut malapposition were 34.9% (range 18.2–57.1%) and 7.0% (range 3.3–13.8%), respectively. Besides, there were the highest rates of tissue protrusion and stent strut malapposition in Case 4. Most of the tissue protrusion was smooth tissue protrusion. Small dissection may appear sometimes (Cases 2 and 4). Two patients underwent follow-up OCT examination to evaluate the pattern of neointimal hyperplasia in RICS patients (Cases 3 and 4). There were homogeneous signal-rich neointima and signal-poor regions around stent struts in both follow-up OCT images. The neointima burden of Cases 3 and 4 was 31.6 and 48.9%, respectively. Microvessels were observed in the thicker 6-month neointima of Case 4. Detailed data are summarized in Table 2.

### Case 1

**FIGURE 1:**
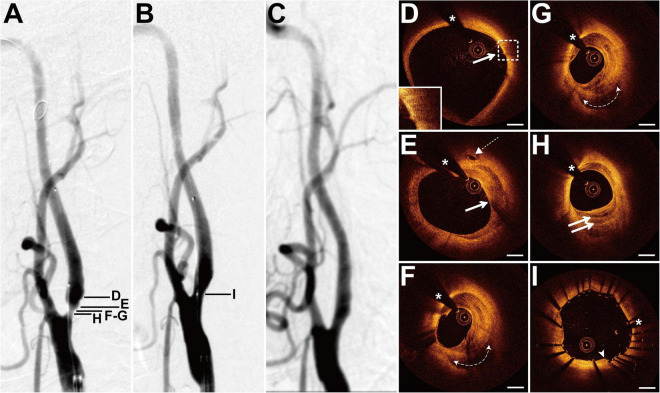
DSA and OCT findings of the LICA lesion. Case 1. **(A)** The angiogram before stenting showed severe stenosis at the LICA sinus. **(B)** The angiogram after stenting. **(C)** The angiogram at a 4-month follow-up revealed no in-stent restenosis. **(D)** The macrophage accumulations (arrow) at 2 o’clock. **(E)** The microvessel (dashed arrow) at 12 o’clock and the heterogeneous signal-rich tissue (arrow) at 12–6 o’clock. **(F,G)** Adjacent OCT images. **(G)** The image with the minimum lumen. The heterogeneous tissue occupied 3/4 of the vessel and the irregular signal-poor tissue (dashed curve with arrows) accumulated at 5–7 o’clock. **(H)** The onion-like structure (double arrow). **(I)** Post-interventional OCT image with the minimum lumen. Stent struts were well apposed and there was smooth tissue protrusion (arrowhead) between stent struts. Scale bars represent 1 mm. Asterisks denote guide-wire artifact. DSA, digital subtraction angiography; LICA, left internal carotid artery; OCT, optical coherence tomography.

The patient presented with a history of NPC 4 years had been treated with cervical radiotherapy (70 Gy) and chemotherapy (cisplatin, docetaxel, and bevacizumab). The patient presented with dizziness for 2 months. DSA showed 70% of stenosis at the left ICA (LICA) sinus (Figure 1A). The patient underwent balloon pre-dilatation (5 × 30 mm) and stent implantation (Acculink, 7–10 × 40 mm). The residual stenosis was 20% (Figure 1B). The 4-month follow-up DSA detected that there was no in-stent restenosis (Figure 1C). Pre-interventional OCT examination of the distal lesion with mild stenosis revealed macrophage accumulations at 2 o’clock, and there was no clear three-layered vessel structure (Figure 1D). More proximally, nearly half of the vessel was occupied by a heterogeneous signal-rich tissue located close to the luminal surface (Figure 1E). There was a clear demarcation between the tissue and the outer fibrous tissue. Moreover, there was a microvessel at 12 o’clock (Figure 1E). Nearly three-quarters of the vessel was occupied by the heterogeneous tissue at the minimum lumen, and there was irregular signal-poor tissue accumulating at 5–7 o’clock (Figures 1F,G). At the proximal site of the lesion, there were multiple layers of heterogeneous signal-rich tissue, forming the onion-like structure (Figure 1H). The post-interventional OCT evaluation revealed excellent stent strut apposition and smooth tissue protrusion at the image with the minimum lumen (Figure 1I).

### Case 2

**FIGURE 2:**
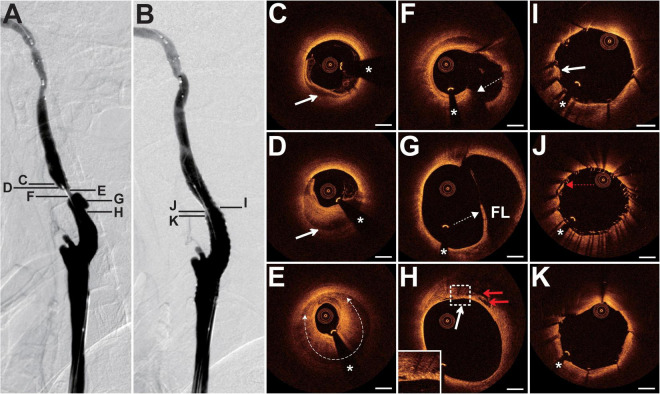
DSA and OCT findings of the LICA lesion. Case 2. **(A)** The angiogram before stenting showed severe stenosis at the LICA C1 segment. **(B)** The angiogram after stenting. **(C)** The intramural hematoma (arrow) at 5–9 o’clock. **(D)** The intramural hematoma (arrow) occupied half of the vessel. **(E)** The intramural hematoma (dashed curve with arrows) expanded to nearly three-quarters of the vessel at the minimum lumen. **(F)** The intimal tear (dashed arrow) at 5 o’clock. **(G)** The double-lumen (the true lumen and the false lumen [FL]) and the floating intimomedial flap (dashed arrow) were identified. **(H)** The cavity formations (red arrows) and the macrophage accumulations (arrow). **(I–K)** Post-interventional OCT images. **(I)** The smooth tissue protrusion (arrow). **(J)** The residual small dissection (red dashed arrow). Image **(J)** corresponds to image **(F)**. Image **(K)** represents the image with the minimum lumen. Stent struts were well apposed. Scale bars represent 1 mm. Asterisks denote guide-wire artifact. DSA, digital subtraction angiography; LICA, left internal carotid artery; OCT, optical coherence tomography.

The patient presented with a history of NPC 8 years had been treated with cervical radiotherapy (70 Gy) and chemotherapy. The patient presented with paroxysmal vertigo for 3 days. Magnetic resonance imaging (MRI) revealed acute cerebral infarction in the right frontal lobe, occipital lobe, and semi-oval center. DSA showed 80% stenosis with an irregular surface at the LICA C1 segment (Figure 2A) and occlusion at the left external carotid artery origin. The patient underwent balloon pre-dilatation (4 × 30 mm) and stent implantation (Precise, 8 × 40 mm) at the LICA lesion. The residual stenosis was 20% (Figure 2B). Pre-interventional OCT examination of the distal lesion revealed that the crescent-shaped material separated the intima from the outer vessel wall at 5–9 o’clock (Figure 2C). More proximally, nearly half of the vessel was occupied by the relatively homogeneous signal-rich material with variable attenuation that separated the intima from the outer vessel wall, indicating intramural hematoma (Figure 2D). The intramural hematoma expanded to almost three-quarters of the vessel at the minimum lumen (Figure 2E). In addition, the intimal tear at 5 o’clock, intimomedial flap floating, and double-lumen formation were well visualized by OCT (Figures 2F,G), demonstrating dissection. Multiple cavity formations and macrophage accumulations were detected at the proximal normal-appearing vessel (Figure 2H). The post-interventional OCT evaluation disclosed excellent stent strut apposition, smooth tissue protrusion, and a residual small dissection (Figures 2I–K). The dissection corresponded to the intimal tear mentioned above (Figure 2J).

### Case 3

**FIGURE 3:**
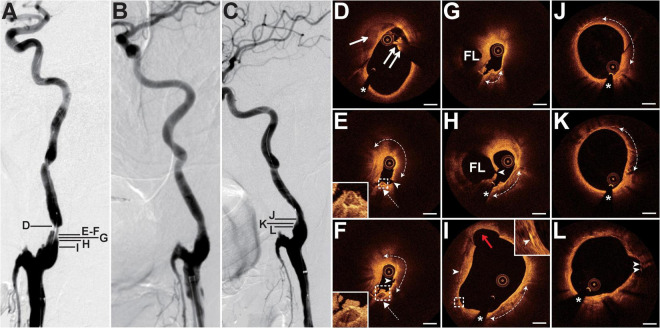
DSA and OCT findings of the LICA lesion. Case 3. **(A)** The angiogram before stenting showed severe stenosis with an irregular surface at the LICA C1 segment. **(B)** The angiogram after stenting. **(C)** The angiogram at a 13-month follow-up revealed no in-stent restenosis. **(D)** The crescent-shaped intramural hematoma (arrow) and the intraluminal thrombus (double arrow). **(E,F)** Adjacent OCT images. Image **(E)** represents the image with the minimum lumen. The lipid-rich plaque (dashed curve with arrows) with plaque rupture (arrowheads) and adjacent ruptured calcific nodule (dashed arrows) with overlying thrombus were detected. **(G,H)** The double-lumen and the fenestration (arrowhead) between the true lumen and the false lumen (FL). The calcification (dashed curve with arrows) with overlying thrombus. **(I)** The cavity formation (red arrow), cholesterol crystals (arrowheads), and calcification (dashed curve with arrows) were observed. **(J–L)** Thirteen-month follow-up OCT images. Image **(J)** represents the image with the minimum lumen. Homogeneous neointima and signal-poor regions around stent struts (dashed curve with arrows) were detected. There were uncovered stent struts (arrowheads) in the cavity. Image **(L)** corresponds to image **(I)**. Scale bars represent 1 mm. Asterisks denote guide-wire artifact. DSA, digital subtraction angiography; LICA, left internal carotid artery; OCT, optical coherence tomography.

The patient presented with a history of NPC of 36 years had been treated with cervical radiotherapy. The patient presented with recurrent weakness and numbness of the right limb, and slurred speech for 1 month. MRI revealed acute cerebral infarction in the bilateral frontal and parietal lobes. DSA showed 90% of stenosis with an irregular surface at the LICA C1 segment (Figure 3A) and occlusion at both the left external carotid artery and the right CCA (RCCA) origin. Balloon pre-dilatation (4 × 30 mm), stent implantation (Acculink, 9 × 40 mm), and balloon post-dilatation (5 × 20 mm) were performed at the LICA lesion. The residual stenosis was 20% (Figure 3B). The 13-month follow-up DSA showed no in-stent restenosis (Figure 3C). Pre-interventional OCT examination of the lesion revealed that the crescent-shaped material separated the intima and the outer vessel wall at 9–1 o’clock, indicating intramural hematoma (Figure 3D). In addition, intraluminal thrombus was observed and shadowed the underlying vessel wall (Figure 3D). At the minimum lumen, there was a ruptured lipid-rich plaque and a ruptured calcific nodule with an overlying thrombus next to the intramural hematoma (Figures 3E,F). More proximally, the double-lumen with the fenestration communicating the true lumen and the false lumen was identified as the sign of dissection (Figures 3G,H). In addition, the calcification with overlying thrombus (Figures 3G,H) and the ruptured calcific nodule mentioned above were consecutive. The length of the calcification reached 6.8 mm. Cholesterol crystals and cavity formations due to intimal disruption were frequently detected at the proximal site of the lesion (Figure 3I). The 13-month follow-up OCT examination revealed mild in-stent neointimal hyperplasia (Figures 3J–L). The neointima was homogeneous signal-rich, and there were signal-poor regions near the stent struts (Figures 3J,K). Some struts in the cavity mentioned above were not covered by the neointima (Figure 3L).

### Case 4

**FIGURE 4:**
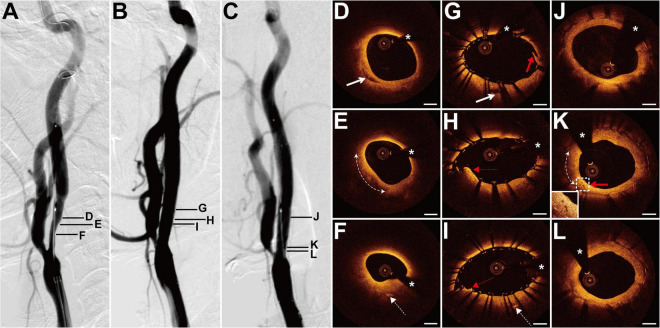
DSA and OCT findings of the RICA lesion. Case 4. **(A)** The angiogram before stenting showed a long lesion with severe stenosis at the RICA sinus and C1 segment. **(B)** The angiogram after stenting. **(C)** The angiogram at a 6-month follow-up revealed no in-stent restenosis. **(D)** The focal signal-poor tissue (arrow). **(E)** The lipid-rich plaque (dashed curve with arrows). **(F)** The image with the minimum lumen. The cholesterol crystals (dashed arrow) at 6 o’clock. **(G–I)** Post-interventional OCT images. **(G)** The small dissection (red arrow) at the cross section with the focal signal-poor tissue (arrow). Image **(G)** corresponds to image **(D)**. Image **(H)** represents the image with the minimum lumen. The smooth tissue protrusion (red dashed arrow) between stent struts was detected. **(I)** The irregular tissue protrusion (red dashed arrow) at the cross section with the cholesterol crystals (dashed arrow) was observed. Image **(I)** corresponds to image **(F)**. **(J–L)** Six-month follow-up OCT images. Image **(J)** corresponds to image **(G)**. Image **(L)** represents the image with the minimum lumen. The homogeneous signal-rich neointima, signal-poor regions around stent struts (dashed curve with arrows), and microvessels (red arrow) were visualized. Scale bars represent 1 mm. Asterisks denote guide-wire artifact. DSA, digital subtraction angiography; OCT, optical coherence tomography; RICA, right internal carotid artery.

The patient presented with a history of cervical esophageal carcinoma 4 years had been treated with cervical radiotherapy (70 Gy) and chemotherapy (nedaplatin, docetaxel, and raltitrexed). The patient presented with slurred speech and paroxysmal unconsciousness for 3 days. MRI revealed acute cerebral infarction in the left frontal and temporal lobes. DSA showed a long lesion with 70% stenosis at both the right ICA (RICA) sinus and C1 segment (Figure 4A) and occlusion at the LICA sinus. The patient underwent balloon pre-dilatation (5 × 30 mm) and stent implantation (Protégé, 8 × 60 mm) at the RICA lesion. The residual stenosis was 20% (Figure 4B). The 6-month follow-up DSA showed no in-stent restenosis (Figure 4C). Pre-interventional OCT examination of the lesion disclosed focal signal-poor tissue in the fibrous tissue (Figure 4D). More proximally, there was a lipid-rich plaque with a thick fibrous cap at 6–10 o’clock (Figure 4E). At the minimum lumen, the intima thickened with mainly fibrous tissue and linear signal-rich cholesterol crystals were detected at 6 o’clock (Figure 4F). The cholesterol crystals were detected at 10 adjacent cross sections. The post-interventional OCT evaluation disclosed the small dissection, smooth tissue protrusion, and irregular tissue protrusion at the stented segment (Figures 4G–I). Besides, its rates of tissue protrusion and stent strut malapposition were the biggest among the four patients. The 6-month follow-up OCT examination revealed evident in-stent neointimal hyperplasia, signal-poor regions around stent struts, and neovascularization (Figures 4J–L). Stent struts were all covered by the neointima, and the small dissection was healed (Figure 4J). There were some microvessels near the signal-poor regions (Figure 4K). The neointima with the minimum lumen was fibrotic and homogeneous signal-rich (Figure 4L).

### Case 5

**FIGURE 5:**
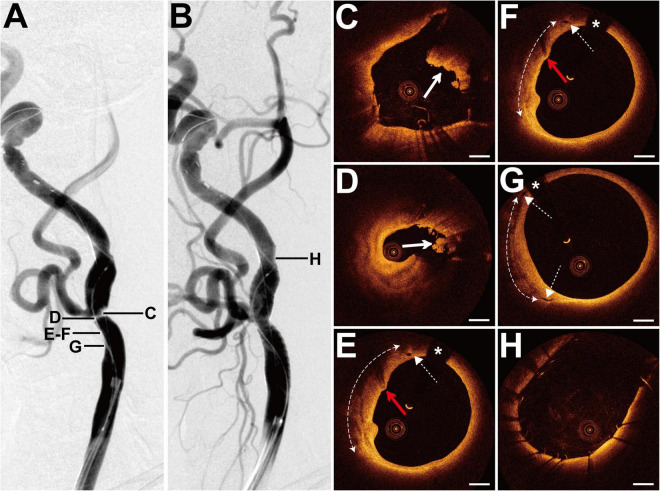
DSA and OCT findings of the LCCA lesion. Case 5. **(A)** The angiogram before stenting showed severe stenosis at the bifurcation of the LCCA. **(B)** The angiogram after stenting. **(C)** The intraluminal thrombus (arrow). **(D)** The fibrous plaque with the intraluminal thrombus (arrow) at the minimum lumen. **(E–G)** Images **(E,F)** are adjacent OCT images. The lipid-rich plaque (dashed curve with arrows) with macrophage accumulations (red arrows) and adjacent microvessels (dashed arrows) were detected. **(H)** Post-interventional OCT image. Most stent struts were well apposed, and some of the vessel walls were out of the imaging range. Scale bars represent 1 mm. Asterisks denote guide-wire artifact. DSA, digital subtraction angiography; LCCA, left common carotid artery; OCT, optical coherence tomography.

The patient presented with a history of cervical lymphoma for 15 years had been treated with cervical radiotherapy and chemotherapy. The patient presented with recurrent vision loss in the right eye for more than 1 year and paroxysmal vertigo for 2 months. DSA showed 70% stenosis at the bifurcation of the left CCA (LCCA) (Figure 5A) and occlusion at the RCCA origin. Balloon pre-dilatation (4 × 30 mm), stent implantation (Precise, 8 × 40 mm), and balloon post-dilatation (6 × 30 mm) were performed at the LCCA lesion. The residual stenosis was 20% (Figure 5B). Pre-interventional OCT examination of the lesion revealed the intraluminal thrombus floating at 12–3 o’clock (Figure 5C). There was a fibrous plaque with the intraluminal thrombus at the minimum lumen (Figure 5D). A lipid-rich plaque with macrophage accumulations was visualized at the proximal site of the lesion (Figures 5E,F). In addition, there was a microvessel connecting with the edge of the lipid plaque. More proximally, the lipid plaque with the neovascularization still existed (Figure 5G). The length of the lipid plaque reached 5.5 mm. The post-interventional OCT evaluation disclosed fine stent strut apposition, and some of the vessel walls were out of the imaging range (Figure 5H).

## Discussion

The goal of this study was to utilize OCT to reveal the morphological characteristics of RICS and the stent–vessel interactions in patients with previous cervical radiotherapy. In this pilot study, several morphological characteristics of RICS were exhibited by OCT, such as heterogeneous signal-rich tissue, dissection, and advanced atherosclerosis. Based on the post-interventional OCT findings, the mean rates of tissue protrusion and stent strut malapposition were 34.9% (range 18.2–57.1%) and 7.0% (range 3.3–13.8%), respectively. Follow-up OCT examinations of the two patients revealed homogeneous signal-rich neointima and signal-poor regions around stent struts. Microvessels were observed in the thicker 6-month neointima where the neointima burden reached 48.9%.

Although the lesion characteristics of RICS were studied through various imaging tools, the mechanism of RICS has been undetermined. Zou et al. (2013) applied DSA and discovered that there was more bilateral severe stenosis at the CCA/ICA (18% vs. 6%) and dissections (20% vs. 3%) in patients with symptomatic occlusive radiation vasculopathy than in patients with severe symptomatic carotid stenosis. In line with the research by Zou, bilateral severe stenosis at the CCA/ICA and dissection were frequently present in this study. As for the plaque composition, multidetector row computed tomography showed a significant increase in the carotid artery plaque volume and the percentage of fatty plaque component at 2 years after radiotherapy (Anzidei et al., 2016). Moreover, the carotid ultrasound scanning revealed more hypoechoic plaque (9% vs. 0%) in irradiated NPC patients (4–11 years after radiotherapy) than in non-irradiated patients (Lam et al., 2002). Hypoechoic plaque may indicate lipid plaque or intraplaque hemorrhage and represent vulnerable plaque. However, Fokkema et al. (2012b) compared the histopathological features of RICS (1.8–24 years after radiotherapy) with atherosclerotic-induced carotid stenosis and discovered that RICS had less infiltration of macrophages and a smaller lipid core size, indicating a more stable plaque in RICS. The tumor type, the radiation dose, the time interval from radiotherapy, concomitant chemotherapy (cisplatin), and the existing cardiovascular risk factors were associated with RICS (Cheng et al., 1999; Dorth et al., 2014; Twitchell et al., 2018). We supposed that the heterogeneity of tumor types and time intervals from radiotherapy in these two research works may explain the opposite results.

The heterogeneous lesion characteristics of RICS were also observed by OCT in our study. As for the NPC patients (Cases 1–3) with increasing time intervals from radiotherapy, heterogeneous signal-rich tissue, dissection, and advanced atherosclerosis were identified in sequence. As far as we know, OCT has been applied to reveal the features of carotid atherosclerotic stenosis and the stent–vessel relationship after stenting. As for the features of carotid stenosis, advanced atherosclerosis such as ruptured lipid-rich plaque, ruptured calcific nodule, neovascularization, and thrombosis were both observed in carotid atherosclerotic stenosis (Yoshimura et al., 2011, 2012; Jones et al., 2014; Yang et al., 2021) and RICS. Interestingly, heterogeneous signal-rich tissue and onion-like structure were observed in our study. Heterogeneous signal-rich tissue may represent layers of distinct collagen types or organized thrombi and be explained by prior rupture and healing (Shimokado et al., 2018; Vergallo and Crea, 2020). As we know, radiation can damage the vascular endothelial cell and promote thrombosis (Zheng et al., 2020). Furthermore, radiation damage to the vasa vasorum may cause loss of the elastic fibers and smooth muscle fibers and lead to dissection. With the application of OCT, we can clearly identify the various lesion features of RICS and may guide individualized treatment in the future.

Carotid artery stenting and carotid endarterectomy were both effective treatments for reducing the risk of stroke in patients with severe carotid stenosis. On account of the fact that previous cervical radiotherapy could cause absent tissue planes in the carotid artery wall and poor tissue healing, CAS has been assumed as a less invasive alternative to reduce the risk of complications with surgery (Kernan et al., 2014). A meta-analysis compared the outcome of CAS with carotid endarterectomy in patients with RICS and discovered that CAS had higher rates of restenosis and late cerebrovascular adverse event (Fokkema et al., 2012a). In addition, Yu et al. reported that the rates of in-stent restenosis in patients with RICS were significantly higher than in patients with carotid atherosclerotic stenosis (26% vs. 4%) (Yu et al., 2014). The stent–vessel interactions in RICS have not yet been studied. In this study, the mean rates of tissue protrusion and stent strut malapposition were 34.9 and 7.0%, respectively. With the high rates of tissue protrusion and stent strut malapposition in Case 4, its 6-month neointima burden reached 48.9% and neovascularization was observed. The stent strut malapposition may increase the risk of stent thrombosis (Ng et al., 2017), and irregular tissue protrusion may be associated with target lesion revascularization (Soeda et al., 2015). Whether the rates of tissue protrusion and stent strut malapposition affect the stent restenosis in irradiated patients needs further exploration.

This study has some limitations. First, it is a single-center study with small sample size. Future work should increase the sample size to compare RICS with carotid atherosclerotic stenosis by OCT. Second, although various morphological characteristics of RICS were detected in this study, consistent features of RICS need future exploration. Third, more post-interventional OCT and follow-up OCT evaluations are required to investigate the mechanism of stent restenosis in irradiated patients.

## Conclusion

The morphological features of RICS were heterogeneous, including heterogeneous signal-rich tissue, dissection, and advanced atherosclerosis. Stenting was successful in all 5 patients with severe radiation-induced carotid stenosis. One patient, with high rates of tissue protrusion and stent strut malapposition immediately after stenting, received in-stent neointimal hyperplasia at a 6-month follow-up.

## Data Availability Statement

The original contributions presented in the study are included in the article/supplementary material, further inquiries can be directed to the corresponding author/s.

## Ethics Statement

The studies involving human participants were reviewed and approved by the Jinling hospital’s Ethics Committee. The patients/participants provided their written informed consent to participate in this study.

## Author Contributions

XX and FH participated in the study design, collection, interpretation, analysis of data, and writing of the manuscript. XS and RL participated in the collection, interpretation, and analysis of data. YH, ML, FW, QY, and WZ participated in the analysis of data and revising of the manuscript. XL and RY participated in the study design, collection, interpretation, analysis of data, and revising of the manuscript. All authors contributed to the article and approved the submitted version.

## Conflict of Interest

The authors declare that the research was conducted in the absence of any commercial or financial relationships that could be construed as a potential conflict of interest.

## Publisher’s Note

All claims expressed in this article are solely those of the authors and do not necessarily represent those of their affiliated organizations, or those of the publisher, the editors and the reviewers. Any product that may be evaluated in this article, or claim that may be made by its manufacturer, is not guaranteed or endorsed by the publisher.
